# Suits against Surgeons

**Published:** 1879-03

**Authors:** George M. Blake

**Affiliations:** Dansville, N. Y.; A. A. Mich.


*ART. IV.—Suits against Surgeons.* By GEO. M. BLAKE, M. D., Dansville, N. Y.

Suits against surgeons for alleged want of skill or negligence are becoming of more and more frequent occurrence. There is a tendency at the present time to impose upon this class of professional men perilous obligations, beyond the requirements of the law. It is the theory of our law to submit the decision of all questions of fact to a jury, and while the court may lay down in this class of cases, as in others, for the guidance of the jury, the legal principles bearing upon them, there is always left for the jury the question of whether the surgeon did or did not possess reasonable skill, or did or did not exercise that skill in the particular case, and the jury, aided by the plaintiff's witnesses, who are generally possessed of remarkably acute hindsight as to what ought to have been done, almost invariably judge the surgeon by the result in the particular case, without reference to anything else. So that there is as much truth as satire in the saying of a witty Frenchman that "A surgeon was an unfortunate gentleman, who was expected to perform a miracle or so, daily." Add to this tendency of a jury to judge by the event the fact that the intelligent classes of our communities shun jury duty, and that juries are come to be regarded, with some showing of truth, as made up of the ignorant and prejudiced, and it will readily be seen that though the surgeon is tried by a jury of his political peers, he is also damnified by a jury utterly unfit to decide this or any other class of cases wherein questions of science are mooted. That a surgeon who maltreats a case submitted to his hands for cure, should pay for the consequences of his ignorance, and so far as money can do it, should indemnify his injured patient, is no more than just, but it is notorious that where one case of this kind arises, there are ten where the surgeon has not been at fault at all, where, though his treatment may not have resulted in cure, he still ought not to be subjected to the annoyance of defending a suit. The causes for the multiplicity of these cases are several. The plaintiff is under no obligation to secure the costs in case of his defeat, and should he prove a pauper, as many of them are, the surgeon is robbed of just so much time, cost and anxiety as the case produces, and be his defence and justification ever so complete, he has no redress. These cases brought for pauper clients by unscrupulous attorneys on speculation, make up the majority of malpractice suits. Another operative cause tending to increase their number, is the well understood jealousy of surgeons one for another, and of those of one school for those of another, of Homœopath for Allopath, and vice versa. Then, too, a malpractice suit is such an easy and cheap way for a man who fancies himself aggrieved, to get his revenge upon a surgeon, that suits cooked up out of spite, are not unknown. It is not my intention, however, to go into the causes of these suits further than to show how peculiarly liable surgeons are to unjust suits for alleged malpractice, and unless they keep standing witnesses over the patient all the time to see that he does not molest or tamper with the bandages or the like, and so produce the very result on which he grounds his suit, how impossible it is to prove to the satisfaction of a jury that the surgeon was not at fault. What I do desire, is to aggregate, so far as I may, the rules of law settled by the authorities, which especially bear upon this subject. A surgeon may be at fault in one of two ways—he may wholly lack the required skill, or, having that skill, he may negligently fail to exercise it. Usually both faults are charged in the complaint, and in practice it is somewhat difficult to separate them. The degree of skill which the law requires, is the same, neither more nor less, than it demands of attorneys and other men who hold themselves out to the public as possessed of peculiar skill or knowledge. It demands that amount of skill which is possessed by the average ordinary professor or practitioner of that art or science.

"The duties and responsibilities of all these classes, as those of lawyers and physicians are governed by the same general rules." (1 Bour. Inst. 403.)

"The person undertaking to act as a surgeon engages that he possesses that reasonable degree of learning and skill which is ordinarily possessed by the professor of the same art or science, and which is ordinarily regarded by the community, and by those conversant with the employment, as necessary to qualify him to engage in such business." (Per Allen, J., in Carpenter *v.* Blake, N. Y. Ct. of App's, partially reported, 50 N. Y. 696.

"He is not responsible for want of success unless it is proved to result from want of ordinary skill, or want of ordinary care and attention. He is not presumed to engage for extraordinary skill, or for extraordinary diligence." (Per Bell, J., Leighton *v.* Sargent, 7 Fost. N. H. 460.) (Story Bailments, § 433.) (L. I. Brougham, in Purves *v.* Laudell, 12 Clark & Finnelly 91.) (Sherman & Redfield, Negligence, § 434 and note 4.)

Some of the authorities above cited are in the cases of suits against attorneys, but certainly if there is any difference, it must be in the application of the most favorable rules to the duties of urgeons. They have usually no opportunity to consult books, and are often compelled to act suddenly with little or no time for reflection, and amidst great excitement and distress. The condition of their patients is often very dissimilar, one tough, robust and strong nerved, another an hysterical female, one surrounded with intelligent aid, and nurses to understand and carry into effect the directions of the surgeon, the other ignorant, careless and deceitful, who would take off his bandages if they pained him, to replace them only when he expected the doctor's visit. In all these cases the doctor must use his own best judgment as to the means and appliances best fitted, not only to the injury, but also to the subject and choose which of the two or more methods is the best under all the surroundings for the often impatient patient.

"The standard of skill may vary according to circumstances, and may be different even in the same state or country. In country towns and unsettled portions of the country remote from cities, physicians, though well informed in theory, are but seldom called upon to perform difficult operations in surgery, and do not enjoy the opportunities of daily observation and practice which large cities afford. It would be unreasonable to exact of one in such circumstances that high degree of skill which an extensive and constant practice in hospitals or large cities would imply a physician to be possessed of." (Sherman & Redfield, Negligence, § 436.)

Where want of skill is made one of the material allegations of the complaint, evidence of the defendant's skill, education, general good reputation and standing in his profession is admissible in rebuttal of the allegations. "Where the issue is made upon the possession of skill, not merely the use of it, the defendant may introduce evidence of his general skill, and where there is much doubt as to the skillfulness of his treatment of the particular case, evidence of his general skill will be *material upon all the issues* of the cause, for if he had skill it is natural to suppose he used it." (McCaudles *v.* McWha, 25 Pa. State, 95.)

"Where the declaration against a surgeon alleged that the plaintiff sustained injury from the want of skill and mere neglect of the surgeon in the treatment of a fracture, evidence that the defendant had received a good surgical and medical education, and was a regularly educated and skillful surgeon, was admissible because it tended to disprove a material allegation of the declaration." (Per Bell, J. Supra.)

"It was manifest error, and prejudicial to the defendant to charge that it was immaterial whether the defendant was or was not reputed to be, or was or not a skillful surgeon. This was one of the material issues of the case. If he had not competent skill he was censurable for holding himself out to the public as possessed of that skill, and the jury would properly hold him strictly accountable for the consequences of his acts. He would not be entitled to the benefits which would enure to the skillful surgeon from an error of judgment or mistake in the means and appliances at command of the expert. Not having skill or experience he could have no reasons, and could exercise no discretion or choose between different methods, and having adopted a process which was not successful to the exclusion of one which might and probably would prove successful, he cannot fall back for protection upon that which serve as a shield to the skilled operator. That having skill and knowledge to choose between two or more courses of treatment in the exercise of his judgment, and with knowledge of all, he honestly and fairly chose the one to the exclusion of the other." (Opinion. Allen, J. Supra.)

Any evidence then calculated to repel the inference of ignorance and unskillfulness—to show that the surgeon is a man of suitable education and acquirements for the safe practice of his profession, is competent and proper. Such evidence changes the whole position of a case, for if a jury is satisfied that the surgeon had proper knowledge and skill, the only question then left to them is, whether he adopted the course of his treatment from mistake, mere error of judgment, for which he is not liable, or from negligence and want of ordinary care which constitutes the second ground of the surgeons liability.

"The law implies an undertaking on the part of every medical practitioner, that he will use ordinary care and skill in his practice, and will hold him liable for *gross* carelessness or unskillfulness." (Bowman *v.* Wood, 1 Green, Iowa, 441.)

"A surgeon undertakes that he will use reasonable and ordinary care and diligence in the execution of his skill and the application of his knowledge to accomplish the purpose for which he is employed." (Allen, J., Supra.)

As the law requires only ordinary skill, so it only requires ordinary care and diligence, but upon this point of ordinary care it might be made a question whether a medical man is not really bound to apply extraordinary care because his charge relates to the lives and health of his patients, which are of unequalled importance and interest to them. But there is no pretense that the physician is bound to any other rule in this respect than that which governs all classes of men engaged in works and services requiring skill—the rule of ordinary care and diligence. "He is not responsible for errors of judgment or mere mistakes in matters of reasonable doubt and uncertainty. * * * * To charge a physician or surgeon with damages, on the ground of unskillful or negligent treatment of his patients case, it is never enough to show that he has not treated his patient in that mode, nor used those measures which, in the opinion of others, even medical men, the case required, because such evidence tends to prove errors of judgment, for which the defendant is not responsible as much, as the want of reasonable care and skill for which he may be responsible alone, it is not evidence of the latter, and therefore the party must go farther and prove by other evidence that the defendant assumed the character and undertook to act as a physican without the education, knowledge and skill which entitled him to act in that capacity. * * * * They should be chargeable with the consequences of mere error only, where such errors could not have arisen, except from want of reasonable skill or diligence." (Bell, J., Supra.)

A surgeon engages "To use his best judgment in the application of his skill, and the exertion of his diligence." (Allen, J., Supra.)

The surgeon is called upon to use his own best judgment, not that of any authority, hence if he believes some course not set down in the books, will be of benefit to his patient in a particular case, and has good reason for the faith that is in him, he is at liberty to use it, as he is also to choose when the authorities differ, or set forth several modes of treatment. But if the settled practice and surgical authority allow but one course of treatment in a case, then any material departure from such course might properly be regarded as the result of want of care and attention, and such departure, if made in a case which is not doubtful or uncertain, is made at the peril of the surgeon. (Patten *v.* Wiggan, 51 Maine, 594. Ritchey *v.* West, 23 Illinois, 385; Bowman vs Wood, 1 Green, Iowa, 441 ; Landon *v.* Humphrey, 9 Connecticut, 209.

The party seeking to charge the surgeon with want of skill or want of care, or failure to exercise his best judgment, holds the affirmative, and must establish the surgeon's default by satisfactory proof; the presumption is in his favor. (Sherman & Redfield, Negligence, § 442.)

"Where the plaintiff relies upon non-recovery, or slow recovery, as some evidence of the surgeons unskillfulness or neglect, the defendant is at liberty to prove anything tending to show that the fault was in the patient, not in the treatment."

(McCandles *v.* McWha, Supra), in which case defendant was allowed to show that previous intemperate habits of patient aggravated an accidental injury. The question has been raised as to how long a surgeon was bound to continue in attendance upon his patient in the absence of a special contract, and Sherman & Redfield, in their work on Negligence, lay down a very strict rule. They say a physician has no right to resign charge of a patient before the end of the illness which he undertook to treat. To any one acquainted with the daily exigencies of medical practice, the absurdity of this rule, if it be a rule, is at once manifest. But it seems to be only the dictum of the authors. They support it by no references to decisions by the bench, and it is not the law of New York, as it certainly is not of common sense. Judge Allen, of N. Y., Ct. of Appeals in Carpenter *v.* Blake, gives the rule thus, "A surgeon is not bound, in the absence of a special contract, to attend the patient any longer than the necessities of the case require, and having performed an operation to meet an exigency, he can then withdraw *upon reasonable notice*, leaving the patient to secure other attendants to complete or watch over the cure, and in such case the withdrawing surgeon is only liable for neglect or want of skill up to the time when he committed the case to other hands." The rule in Sherman & Redfield is laid down so strictly because of the parallel instituted between attorneys and surgeons, but on this point the parallel is an unjust one. The employment of attorneys has nothing of the sudden and imperative character of the employment of surgeons, and our common experience teaches that surgeons often, indeed generally in difficult cases, perform the most important operations, and neither give nor expect to give any farther attention to them, and they do this as a matter of right and not of license. Of course they cannot quit in the midst of an operation, or at any time when the case demands their immediate attention, but subject to this limitation I believe the law is as put by Judge Allen, that they are free to cease attendance *upon reasonable notice*, for cannot a patient dismiss a surgeon with or without cause in the absence of agreement, and if one party is not bound, ought not the other be free also? The rules of law laid down by the authorities, the principal of which I have quoted are just to the surgeon. They ask only that which every member of that large and honorable profession willingly concedes to be his duty towards those who employ him. The evil is in the imperfections and prejudices of the twelve specimens of human nature in the jury-box, to whom all questions of fact are submitted. The jury system with all its defects, defects which are inherent in humanity itself, is undoubtedly the best and safest mode of trial known, and we must abide by it.

The only remedy apparent by which the evil effects of its prejudices in these cases can be obviated, is for the "Bench" to impress upon the attention of the jury with more than ordinary care and force, the rules of law governing the surgeons liability.

A. A. Mich., January 26th, 1879.

